# Comparison of the Proseal, Supreme, and I-Gel SAD in Gynecological Laparoscopic Surgeries

**DOI:** 10.1155/2015/634320

**Published:** 2015-02-23

**Authors:** Sanli Mukadder, Begec Zekine, Kayhan Gulay Erdogan, Ozgul Ulku, Ucar Muharrem, Yologlu Saim, Durmus Mahmut

**Affiliations:** ^1^Department of Anesthesiology and Reanimation, Inonu University School of Medicine, Malatya, Turkey; ^2^Department of Biostatistics, Inonu University School of Medicine, Malatya, Turkey

## Abstract

We compared proseal, supreme, and i-gel supraglottic airway devices in terms of oropharyngeal leak pressures and airway morbidities in gynecological laparoscopic surgeries. One hundred and five patients undergoing elective surgery were subjected to general anesthesia after which they were randomly distributed into three groups. Although the oropharyngeal leak pressure was lower in the i-gel group initially (mean ± standard deviation; 23.9 ± 2.4, 24.9 ± 2.9, and 20.9 ± 3.5, resp.), it was higher than the proseal group and supreme group at 30 min of surgery after the trendelenburg position (25.0 ± 2.3, 25.0 ± 1.9, and 28.3 ± 2.3, resp.) and at the 60 min of surgery (24.2 ± 2.1, 24.8 ± 2.2, and 29.5 ± 1.1, resp.). The time to apply the supraglottic airway devices was shorter in the i-gel group (12.2 (1.2), 12.9 (1.0), and 6.7 (1.2), resp., *P* = 0.001). There was no difference between the groups in terms of their fiber optic imaging levels. pH was measured at the anterior and posterior surfaces of the pharyngeal region after the supraglottic airway devices were removed; the lowest pH values were 5 in all groups. We concluded that initial oropharyngeal leak pressures obtained by i-gel were lower than proseal and supreme, but increased oropharyngeal leak pressures over time, ease of placement, and lower airway morbidity are favorable for i-gel.

## 1. Introduction

The use of supraglottic airway devices (SAD) with a gastric emptying tube in gynecological laparoscopic surgeries is growing. In addition to their ease of placement, they have low airway morbidity along with sufficient airway pressure in the trendelenburg position and so they have been determined as an alternative to endotracheal tube [1, 2].

The proseal SAD (LMA Proseal, Laryngeal Mask Company Ltd., Henley-on Thames, UK) is reusable, supraglottic airway device made of silicon and has a gastric emptying tube and inflatable pharyngeal cuff [3, 4]. The supreme SAD (LMA, Laryngeal Mask Company Ltd., Henley-on Thames, UK) is a single-use inflatable airway device with an ellipsoid semihard head made of medical silicon and gastric emptying tube in addition to the ventilation tube. The i-gel SAD (Intersurgical, Wokingham, UK) is a single-use, hard, supraglottic airway device with a mouth stabilizer resistant to biting, a gastric emptying tube, and a noninflatable elastomer structure head. Proseal SAD has an infection risk due to the fact that it can be used multiple times and therefore should be cleaned and sterilized after each use; this also poses a cost disadvantage. Supreme and i-gel, however, are single-use devices and therefore advantageous [5].

The number of studies comparing these techniques is scarce, and so we compared the three SADs with a gastric emptying tube on paralyzed patients who were to undergo gynecological laparoscopic surgery. Our primary objective was to compare the three SADs in terms of oropharyngeal leak pressure. In addition, we examined the safety of these airways by comparing their ease of placement and placement times, the degree to which vocal cords could be seen via fiberoptic bronchoscopy, pH values of secretion on SAD to determine aspiration or regurgitation, and postoperative airway complications.

## 2. Materials and Methods

The study was carried out at the Malatya Turgut Ozal Medical Center after the approval of the Malatya Clinical Studies Ethical Council (2011/188), and written and oral consents of the patients were taken. Clinical trial registration for this study can be found online (clinicaltrials.gov; registration identifier NCT01909297). One hundred and five ASA I-II patients between the ages of 18 and 60 who were to undergo elective gynecological laparoscopic surgery were included in the study. Physical examination of airway, including Mallampati class, thyromental distance, sternomental distance, interincisor distance, lower jaw movement, and head-neck movement, was evaluated prior to the operation as part of a routine preoperative clinical assessment. Exclusion criteria were patient with body weight below 30 kg and with a BMI over 40, SAD which was tried more than three times or when the SAD could not be placed in 120 s, the trial that was planned to deem a failure, those for whom difficult airway (Mallampati class ≥3, inter-incisor distance <3 cm) was expected, those with high gastric regurgitation and aspiration risk, respiratory system pathology, use of H_2_ blockers, and planned operation time exceeding 2 h.

Patients were separated into three groups; proseal, supreme, and i-gel, via the randomized numbers table obtained from http://www.randomization.com/. The SAD dimension was selected according to patient weight without knowing which SAD would be used. For proseal and supreme SAD, 3 was used for weights of 30–50 kg, 4 was used for weights of 50–70 kg, and 5 was used for weights of 70–100 kg. For the i-gel, SAD number 3 was used for 30–60 kg, 4 was used for weights of 50–90 kg, and 5 was used for weights over 90 kg.

Pulse oximeter, electrocardiography, and noninvasive blood pressure, along with standard monitoring operations, were carried out prior to the surgery following a premedication of midazolam iv 0.03 mg kg^−1^. Following a 3 min preoxygenation period, anesthetic induction was provided via intravenous fentanyl 1-2 *μ* kg^−1^, propofol 2-3 mg kg^−1^, and rocuronium 0.6 mg kg^−1^. Mask ventilation was provided until sufficient muscle relaxation was attained. A prelubricated SAD was placed by an experienced anesthetist in accordance with the directions provided by the manufacturing company. Anesthesia maintenance was obtained by end tidal concentration of sevoflurane 2-3% MAC in a 50% oxygen and 50% air mixture. Pressure controlled ventilation (Drager Cato Edition, Lübeck, Germany) adjusted the airway pressure so that the tidal volume was 8–10 mL kg^−1^, the respiratory frequency was 10–16 min^−1^, and EtCO_2_ was 35–45 cm H_2_O. Proseal and supreme SAD cuffs were inflated via a manometer (Rüsch Endotest, Germany) such that the pressure was 60 cm H_2_O. The time between lifting the mask from the face and placing the SAD until the first effective EtCO_2_ graph occurred was recorded. We recorded the total number of insertion attempts. When the SAD was tried more than three times or when the SAD could not be placed in 120 s, the trial was planned to deem a failure and was excluded from the study.

Following the placement of the SAD, lubricant gel was applied 1 cm proximal to the gastric discharge outlet after which the suprasternal notch test [6] was performed (monitoring the pulsatile movement of the gel in the gastric discharge tube proximally when continuous pressure is applied at the cricoid cartilage level); the gastric discharge tube was placed when SAD location was identified to be correct. 14 French gastric discharge tubes were placed in the proseal and supreme SAD, whereas 12 French gastric discharge tubes were placed in the i-gel. The gastric content was aspirated, and the amount was recorded in milliliters. SAD placement was classified according to difficulty using a five-point scale (1 = easy, 2 = not so easy, 3 = difficulty, 4 = very difficult, and 5 = impossible) [7]. The success of the gastric discharge tube placement was also evaluated using a three-point scale (1 = easy, 2 = difficult, and 3 = impossible) [7].

Oropharyngeal leak pressure was measured three times for each patient, once initially just before the start of surgery, once 30 min after the start of surgery (in the trendelenburg position and intra-abdominal area inflated using CO_2_), and once at 60 min of surgery. The auscultation method was used to measure the oropharyngeal leak pressure. The pressure value at the time when a leak sound occurred from the mouth of the patient was recorded, while 3 L of fresh gas flow was sent to the patient and the adjustable pressure valve was fully closed [7]. A maximum pressure of 40 cm H_2_O was allowed during measurement.

A laryngeal image was recorded at 30 min in the trendelenburg position by using a 3.5 mm fiberoptic bronchoscope (Storz, Bavaria, Germany). The fiberoptic bronchoscope was inserted via ventilation tube of the SAD and a classification between 1 and 4 was made according to the visibility level of the vocal cords (1 = cords not seen; 2 = vocal cords and the anterior of the epiglottis seen; 3 = vocal cords and posterior of the epiglottis seen; and 4 = only vocal cords seen) [8].

Heart rate, mean arterial pressure, SpO_2_, and EtCO_2_ values were recorded at basal, after induction, and for every 5 min following placement of SAD. Airway pressure, inspiratory and expiratory tidal pressure difference, and respiratory rate were recorded prior to and after pneumoperitoneum formed.

At the end of surgery, the muscle relaxation effect was reversed using neostigmine 0.04 mg kg^−1^ and atropine 0.02 mg kg^−1^; the SAD was removed when the patient started spontaneous respiration. pH measurement was made at the anterior and posterior surfaces of the pharyngeal region of SAD using a pH-meter (pH-fix 0–14; Macherey-Nagel GmbH & Co. KG, Düren, Germany). Recorded information included any laryngospasm, desaturation (SpO_2_ < 95%), aspiration (fluid in the ventilation tube), bronchospasm, and blood on the SAD upon removal. Sore throat, pain on swallowing, and hoarseness were evaluated by an anesthetist who was independent of the study 1 h after the patient was taken to the recovery unit.

### 2.1. Statistical Analysis

Our primary comparison parameter was oropharyngeal leak pressure. Sample size was based on a pilot we conducted involving 20 SAD proseal insertions that demonstrated a mean ± standard deviation oropharyngeal leak pressure of 25 (3.6) cm H_2_O. To detect a difference of 10%, power analysis at 80% power and the 0.05 level of significance showed that a sample size of 31 patients would be required. We recruited 35 patients for each group.

Statistical analysis was performed using SPSS for Windows version 16.0 (SPSS Inc., Chicago, IL, USA). Continuous variables were reported as mean ± standard deviation. Categorical variables were reported as number (percent). Normality for continuous variables in groups was determined by the Shapiro-Wilk test. One-way analysis of variance (ANOVA), least significant difference test (LSD), Kruskal-Wallis analysis of variance, and Connover test were used for comparison of continuous variables among the studied groups. Pearson chi-square test was used for comparison of categorical variables among studied groups. A value of *P* < 0.05 was considered significant.

## 3. Results

The study was continued with 105 patients and no patients were excluded from the study. The demographic data and airway properties of the patients were given in Table 1. Whereas the initial oropharyngeal leak pressure was lower in the i-gel compared to the proseal and supreme, it was higher at 30 min in the trendelenburg position and at the 60 min of surgery (*P* < 0.001; Table 2). No cuff leak sound was heard outside of the measurement range. We did not allow the intra-abdominal pressure to exceed 15 cm H_2_O. We did not detect an increase in airway pressure above 25 cm H_2_O even at the maximum trendelenburg position.

Success rate in terms of insertion during the first attempt of proseal, supreme, and i-gel was 74.3%, 85.7%, and 94.3%, respectively. According to ease of placement, grade 1 (easy) ratios of proseal, supreme, and i-gel were 60%, 77.1%, and 91.4%, respectively. SAD placement time was shorter in the i-gel group compared to the proseal and supreme groups (*P* < 0.001) (Table 2). The gastric tube aspirate amounts were similar among the groups (*P* = 0.843) (Table 2).

The fiberoptic imaging is shown in Table 3; statistically no significant difference was found among the groups. There were no differences between the three SAD types in terms of airway pressure and ventilation parameters (Table 2).

In terms of hemodynamic values, the mean arterial pressure difference among the groups and the difference of the mean pulse rates were not statistically significant. The pH values of the anterior and posterior face of the SADs were in the range of 5–7 and the difference was not statistically significant (*P* = 0.948). In addition, the lowest pH values were 5 in all groups (Table 2).

Laryngospasm and desaturation were not observed in any patient. Whereas blood contamination was observed in 5 patients (14.3%) in proseal and 6 patients (17.1%) in supreme groups, none was observed in i-gel group. At 1 h postoperation evaluation, sore throat was not observed in the i-gel group but was observed in 9 (25.7%) and 6 (17.1%) patients, respectively, in proseal and supreme groups; this difference was statistically significant (*P* = 0.007). Hoarseness and pain on swallowing were not observed for the i-gel but were present for proseal [1 (2.9%) and 6 (17.1%), resp.] and supreme [4 (11.4%) and 8 (22.9%), resp.] groups; the values were not statistically significant (*P* = 1.67 and *P* = 4.67, resp.).

## 4. Discussion

In our study we found that insertion time was shorter in the i-gel than proseal and supreme SAD. Although the initial oropharyngeal leak pressure was lower in the i-gel, it was greater at 30 min of surgery and at 60 min of surgery than those of the proseal and supreme. Additionally postoperative sore throat, hoarseness, and pain on swallowing were not observed in the i-gel group.

High leak pressure of airway devices enables the safe of ventilation at high airway pressures, such as that occurs in laparoscopic surgery. In our study three measurements were obtained during the surgery; even though the initial oropharyngeal leak pressure was smaller for the i-gel, it was greater in the trendelenburg position and at 60 min of surgery. The reason for this could be that the thermoplastic cuff of the i-gel expands over time due to body temperature, indicating that it has a better safety. In gynecological laparoscopic surgery, Teoh et al. [7] determined that the oropharyngeal leak pressure was 25.0 cm H_2_O for the i-gel and 26.4 cm H_2_O for the supreme. Shin at al. [9] compared i-gel, Proseal, and classical SAD techniques and oropharyngeal leak pressures were determined as 27 cm H_2_O for the i-gel. In both studies, measurements were made only once after the insertion of the supraglottic airway device.

Although we found no difference among the groups in terms of insertion success, we determined that the insertion time was shorter with i-gel compared to both proseal and supreme. Bamgbade et al. [10] achieved first attempt insertion within 5 s in 290 patients and second attempt insertion within 10 s in 8 patients requiring jaw thrust. In another study i-gel was inserted in 4.4 s, while proseal was inserted in 16 s; i-gel is easy to insert because of its shape, contours, firm stem, bite guard, and buccal stabilizer [11]. In addition, we think that the noninflatable cuff of the i-gel leads to its shorter insertion time compared with the supreme and proseal.

Fiberoptic evaluation, which is an indicator of successful insertion of supraglottic airway devices, was carried out once and at the 30 min of the trendelenburg position. There was no difference among the groups in terms of imaging classification. Jun et al. [12] recorded the head position of patients and reported that the fiberoptic image does not change.

The important problems observed in the trendelenburg position are regurgitation and aspiration. One of the indicators of aspiration is the presence of gastric contents into the ventilation tube of the SAD. No patient in this study regurgitated gastric contents into the ventilation tube. Another method used for assessing the gastric regurgitation in the literature is pH measurement [13–15]. In the case series, Gibbison et al. [16] found 1 aspiration and 2 regurgitations among 280 patients with supine position, but they have not studied pH measurement. Gataure and Latto [13] measured the pH of the secretions at the tip of the SAD with pH paper after removal of the device and a value of ≤3 was defined as possible evidence of regurgitation. Similar to that study, we used pH paper following the removal of supraglottic devices. However, we assessed the two part of the device (the anterior and posterior surfaces) because the single measurement may not accurately reflect the actual incidence of regurgitation. For the reason that the lowest pH values were 5 in all groups, we concluded that there was no regurgitation. However, our measurements were done only following the removal of SAD.

Blood contamination, which is an indicator of airway complication, was observed in the proseal and supreme following the removal of the SAD (in 5 and 6 patients, resp.); there was no blood contamination in the i-gel group. Goyal et al. [17] did not find sore throat and hoarseness even though there was blood contamination in all three SADs (i-gel, proseal, and classical); however, they used these techniques on children and nonparalyzed patients, unlike our study. Similar to our findings, Shin et al. [9] did not determine any blood contamination or sore throat in the i-gel group who underwent orthopedic surgery in the supine position. Teoh et al. [7]. compared i-gel and supreme airways in gynecologic laparoscopic surgery and did not find any sore throat, pain on swallowing, and hoarseness but found one blood contamination in the i-gel group and two in the supreme group. The fact that Shin et al. and Teoh et al. obtained results very similar to ours might be due to their use of paralyzed patients. When Uppal et al. [18] compared the i-gel with a tracheal tube, they found 12% blood contamination in relation with the insertion method and ease. Ragazzi et al. [19] compared target-controlled anesthesia with the i-gel and supreme and found one blood contamination in the i-gel group and two in the supreme group. The gel-like cuff minimizes trauma of the airway and neurovascular compression [10].

Our study has some limitations. Fiberoptic bronchoscope was used only once at the 30 min of the trendelenburg position. We do not know whether there will be a change in the image at the end of surgery compared to the beginning of surgery. pH determination was made only once at the end of surgery, and we did not study whether there was any change when pH was monitored in the trendelenburg position.

In conclusion, we have demonstrated in this study that the proseal, supreme, and i-gel SAD provide a safe airway in paralyzed and pressure controlled ventilation administered gynecological laparoscopic surgeries. While initial oropharyngeal leak pressures obtained by i-gel were lower than proseal and supreme, increased oropharyngeal leak pressures over time, ease of placement, and lower airway morbidity are favorable for i-gel.

## Figures and Tables

**Table 1:** Patients airway and surgery characteristics.

	Proseal (*n* = 35)	Supreme (*n* = 35)	I-gel (*n* = 35)
Age, years	31.9 ± 7.0	30.9 ± 7.1	31.1 ± 7.4
Height, cm	161.2 ± 4.8	161.9 ± 5.4	162.0 ± 5.6
Weight, kg	63.6 ± 8.8	67.9 ± 11.2	62.5 ± 10.3
BMI, kg·m^−2^	24.4 ± 2.9	25.8 ± 3.9	23.8 ± 4.1
ASA class I/II	29 (82.9)/6 (17.1)	26 (74.3)/9 (25.7)	33 (94.3)/2 (5.7)
Mallampati class 1, 2	27 (77.1)/8 (22.9)	24 (68.6)/11 (31.4)	28 (80.0)/7 (20.0)
Thyromental distance			
<6.5 cm	18 (51.4)	18 (51.4)	19 (54.3)
>6.5 cm	17 (48.6)	17 (48.6)	16 (45.7)
Sternomental distance			
<12.5 cm	18 (51.4)	18 (51.4)	18 (51.4)
>12.5 cm	17 (48.6)	17 (48.6)	17 (48.6)
Interincisor distance			
<4 cm	7 (20.6)	5 (14.3)	4 (11.4)
>4 cm	28 (80.0)	30 (85.7)	31 (88.6)
Lower jaw movement; yes/no	35 (100.0)	35 (100.0)	35 (100.0)
Head neck movement			
Normal >90°/abnormal <90°	35 (100.0)	35 (100.0)	35 (100.0)
Duration of surgery; min	65,3	64,8	66,1
Type of surgery; *n* (%)			
Laparoscopic hysterectomy	3 (8.6)	3 (8.6)	1 (2.9)
Laparoscopic cystectomy	5 (14.3)	4 (11.4)	1 (2.9)
Diagnostic laparoscopy	17 (48.6)	16 (45.7)	21 (60.0)
Laparoscopic tubal ligation	5 (14.3)	1 (2.9)	3 (8.6)
Laparoscopic myomectomy	5 (14.3)	9 (25.7)	7 (20.0)

Data are presented as number (proportion) or mean ± SD.

**Table 2:** Airway insertion characteristics, oropharyngeal leak pressure, and ventilatory parameters of each group.

	Proseal (n = 35)	Supreme (n = 35)	I-gel (*n* = 35)	P value
SAD size number: 3/4/5	3/26/6	3/19/13	16/19/0	
SAD insertion attempts				
1	26 (74.3)	30 (85.7)	33 (94.3)	
2	8 (22.9)	4 (11.4)	2 (5.7)	
3	1 (2.9)	1 (2.9)	0 (0)	
Reported ease of placement				
1: easy	21 (60.0)	27 (77.1)	32 (91.4)	
2: not so easy	11 (31.4)	7 (20.0)	3 (8.6)	
3: difficult	3 (8.6)	1 (2.9)	0 (0)	
4: very difficult	0	0	0	
5: impossible	0	0	0	
Successful SAD placement time (sec)	12.2 ± 1.2	12.9 ± 1.0	6.7 ± 1.2	<0.001^*^
Ease of gastric tube insertion				
1: easy	27 (77.1)	31 (88.6)	32 (91.4)	0.195
2: difficult	8 (22.9)	4 (11.4)	3 (8.6)	0.208
3: impossible	0	0	0	
Gastric aspiration (mL)	4.0	3.2	3.2	
Oropharyngeal leak pressure (cmH_2_O)				
Initial	23.9 ± 2.4	24.9 ± 2.9	21.0 ± 3.6	0.001^*^
At 30 min	25.0 ± 2.3	25.0 ± 1.9	28.3 ± 2.4	0.001^*^
At 60 min	24.2 ± 2.1	24.8 ± 2.2	29.5 ± 1.2	0.001^*^
Airway pressure: cmH_2_O				
Before pneumoperitoneum	18.7 ± 0.8	18.9 ± 0.7	18.40 ± 0.7	0.106
After pneumoperitoneum	21.4 ± 0.9	21.3 ± 1.1	21.37 ± 1.1	0.081
Intra-abdominal pressure: cmH_2_O	13.4 ± 0.7	13.4 ± 0.8	13.0 ± 0.7	0.051
respiratory rate: min	11.7 ± 0.5	11.6 ± 0.4	11.4 ± 0.5	0.106
Inspiration tidal volume: mL	510.1 ± 66.5	540.4 ± 94.0	500.5 ± 66.7	0.081
Expiration tidal volume: mL	525.7 ± 66.0	557.3 ± 102.5	513.8 ± 69.7	0.072
Inspiration-expiration tidal volume difference: mL	15.6 ± 6.6	14.2 ± 4.3	13.2 ± 4.6	0.163
pH of the anterior face SAD	6-7 (6.1)	5–7 (6.1)	5–7 (6.1)	0.948
pH of the posterior face SAD	5–7 (6.3)	5–7 (6.1)	5–7 (6.5)	0.043

Data are presented as number, number (proportion), mean ± SD, or min–max (mean). ^*^(i-gel compared to proseal and supreme).

**Table 3:** Fiberoptic imaging classification.

	Proseal	Supreme	I-gel
FS ≤ 1	2 (5.7)	0	0
FS ≥ 2	33 (94.3)	35 (100)	35 (100)

Data are presented as number (proportion). Fiberoptic imaging classification ≥2 is a well-placed indicator.
